# Supplementary figures and images for: Inflammasome genes polymorphisms may influence the development of hepatitis C in the Amazonas, Brazil
Source: PLoS One. 2021 Jun 23;16(6):e0253470. doi: 10.1371/journal.pone.0253470 (PMC8221483; doi:10.1371/journal.pone.0253470)

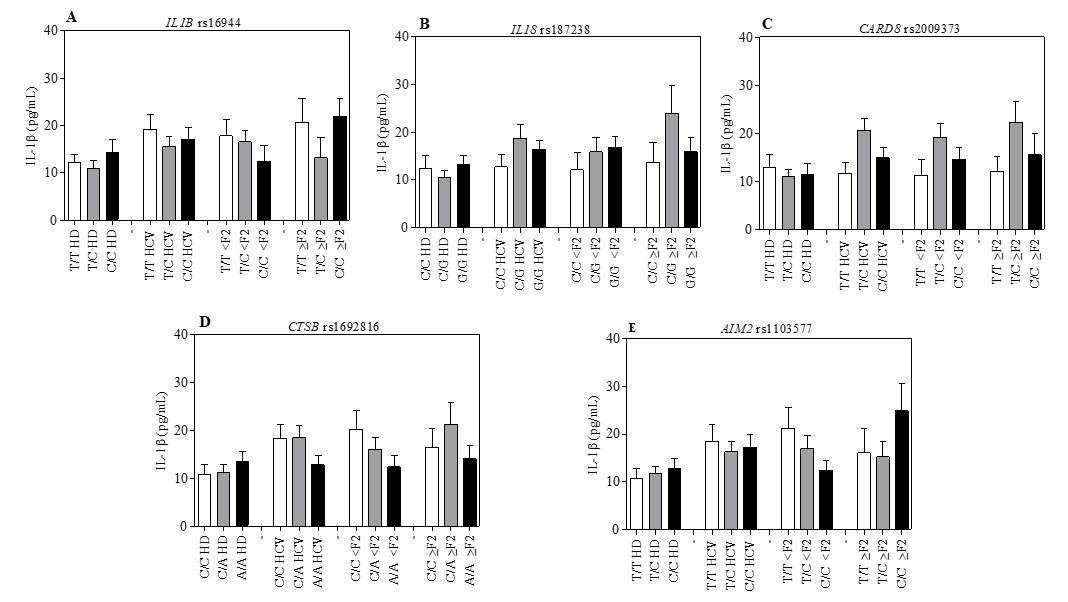

Supplement: S1 Fig — Data are expressed as mean ± standard deviation of circulating concentration (pg/mL) of IL-1β cytokine. Statistical analyzes were performed by ANOVA (nonparametric analysis of variance), with Kruskal-Wallis test, followed by Dunns post-test to compare pairs. (TIF) [file pone.0253470.s001.tif]

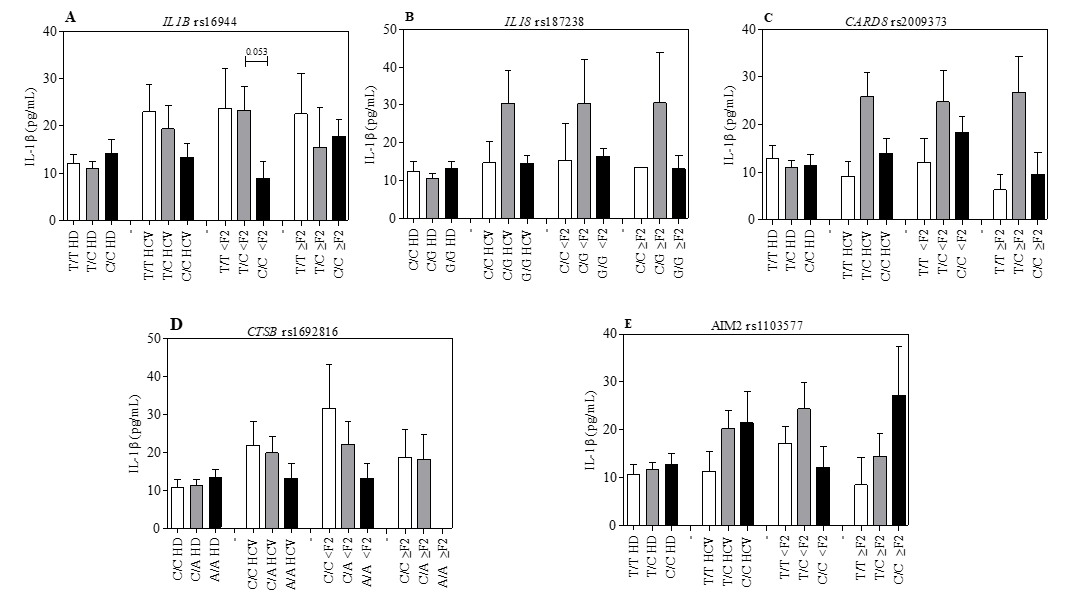

Supplement: S2 Fig — Data are expressed as mean ± standard deviation of circulating concentration (pg/mL) of IL-1β cytokine. Statistical analyzes were performed by ANOVA (nonparametric analysis of variance), with Kruskal-Wallis test, followed by Dunns post-test to compare pairs. (TIF) [file pone.0253470.s002.tif]
