# Supplementary material for: Inflammasome genes polymorphisms may influence the development of hepatitis C in the Amazonas, Brazil
Source: PLoS One. 2021 Jun 23;16(6):e0253470. doi: 10.1371/journal.pone.0253470 (PMC8221483; doi:10.1371/journal.pone.0253470)
Supplement: S1 Table — (DOCX) [file pone.0253470.s003.docx]

**S1 Table.** Sequences of the probes used for Real Time PCR genotyping.

| **Genes** | **SNP Identification** | **SNP and Localization in genome** | **TaqMan probes** |
| --- | --- | --- | --- |
| **CARD8** | rs2009373 | T/C intron | TGCTATCAAAAAAAAAAAAGCATGA[**C/T**]TGTGACACCTGGAAAAAATCTTTTT |
| **CTSB** | rs1692816 | C/A intron | ACACAGCCCTCTTCCCCAGCCCCTC[**A/C**]CCTGCCTGCCCAATCCAGCCCTATT |
| **NLRP3** | rs10754558 | G/C 3'UTR | GACAATGACAGCATCGGGTGTTGTT[**G/C**]TCATCACAGCGCCTCAGTTAGAGGA |
| **AIM2** | rs1103577 | T/C intron | TAAAAGGCACTTCCATTCACTCATT[**C/T**]GCTCAAGCCAGAAAGCCGGGAATCA |
