# Supplementary material for: Inflammasome genes polymorphisms may influence the development of hepatitis C in the Amazonas, Brazil
Source: PLoS One. 2021 Jun 23;16(6):e0253470. doi: 10.1371/journal.pone.0253470 (PMC8221483; doi:10.1371/journal.pone.0253470)
Supplement: S2 Table — (DOCX) [file pone.0253470.s004.docx]

**S2 Table.** Primers and PCR conditions for the studied polymorphisms.

| **SNP Primer sequence, 5’-3’^a^** | **Localization in genome** | **PCR^b^ protocol** | **Restriction Enzymes** | **Enzyme Hybridization Temperature (∘C)** | **Allele, length in bp^d^** |
| --- | --- | --- | --- | --- | --- |
| **IL1β-rs16944**  F:5’-TGGCATTGATCTGGTTCATC-3’  R:5’-GTTTAGGAATCTTCCCACTT-3’ | *-511C/T* | 95˚C for 4 min, 35 x (95˚C for 30 s, 56˚C for 30 s, 72˚C for 30 s), 72˚C for 10 min | *AvaI* | 56°C | T: 304  C: 190 +104 |
| **IL18-rs187238**  F:5’-CACAGAGCCCCAACTTTTTACGGCAGAGAA-3’  R:5’-GACTGCTGTCGGCACTCCTTGG-3’ | *-137G/C* | 95˚C for 4 min, 35 x (95˚C for 30 s, 60˚C for 30 s, 72˚C for 90 s), 72˚C for 10 min | *MboII* | 60°C | C: 155  G: 116 |

^a^ 5’-3’: nucleic acids in the 5’-3’ direction

^b^ PCR: Polymerase Chain reaction

^c^ F and R: Primers forward and reverse

^d^ bp: base pairs.
