# Supplementary material for: Inflammasome genes polymorphisms may influence the development of hepatitis C in the Amazonas, Brazil
Source: PLoS One. 2021 Jun 23;16(6):e0253470. doi: 10.1371/journal.pone.0253470 (PMC8221483; doi:10.1371/journal.pone.0253470)
Supplement: Attachment — Submitted filename: Plos One Report.docx [file pone.0253470.s005.docx]

Article Report

Title: Inflammasome genes polymorphisms may influence the development of Hepatitis C in

the Amazonas, Brazil

**Summary of article:** Diana Mota Toro et al, from Programa de Pós-Graduação em Imunologia Básica e Aplicada, Universidade Federal do Amazonas, Manaus, Amazonas, Brazil. The main focus of this research work is to Hepatitis C and associated polymorphisms of inflammasomes. Author investigated 151 patients with chronic hepatitis C and 206 healthy blood donors’ individuals (HD).

Polymorphisms in the IL1B and IL18 genes were genotyped by PCR-RFLP, while

NLRP3, CARD8, CTSB and AIM2 by RT- PCR.

Serum assay of IL-1β cytokine was performed by ELISA. 84 patients presented mild fibrosis (<F2) and 67 advanced fibrosis (≥ F2). Gene-gene interaction showed that between subgroups of <F2 and ≥F2, individuals homozygous for the T allele of CARD8-rs2009373 and heterozygous for IL18-rs187238 (padj = 0.028), have mild form of fibrosis.

**Comment: Minor revision**

The work is very important in terms of better understanding of association between inflammasomes genes and development of hepatitis C. However, below comments should be address in main content with references to make it more informative and attractive for wide users.

1. Why author choose PCR-RFLP and RT-PCR for polymorphism study. Reason to use PCR-RFLP should be mentioned in main text.
2. Progression of disease depends on multigenic expression of genes in pathway. Why author choose only 6 genes for this study.
3. Genetic background of populations also responsible for false association in the study. Cast and categories along with regional influence might be responsible for various results. Author justify the concern of population stratification if data available about case and control subjects.
4. Sample size is one of challenges in case-control study, however mild vs advanced fibrosis and combined data should be evaluated with statistical corrections for correct p value. Author explain the severity of disease and other gene role in discussion part.
5. How heterogeneity and homogeneity influence the expression of gene and thus the varied clinical out come in HCV.
6. Is there any other study of similar gene and region population in HCV, Kindly mention latest reference to make it for more better understanding.
